# Long-term mortality and incidence of cardiovascular diseases and type 2 diabetes in diabetic and nondiabetic obese patients undergoing gastric banding: a controlled study

**DOI:** 10.1186/s12933-016-0347-z

**Published:** 2016-02-27

**Authors:** Antonio E. Pontiroli, Ahmed S. Zakaria, Ermanno Mantegazza, Alberto Morabito, Alessandro Saibene, Enrico Mozzi, Giancarlo Micheletto, on behalf of the LAGB10 working group

**Affiliations:** Dipartimento di Scienze della Salute, Università degli Studi di Milano, Milan, Italy; Ospedale San Paolo, Milan, Italy; Ospedale San Raffaele, Milan, Italy; Ospedale Policlinico, Milan, Italy; Istituto Clinico Sant Ambrogio, Milan, Italy

**Keywords:** Bariatric surgery, Survival, Adjustable gastric banding, Diabetes mellitus, Cardiovascular disease, Exemptions, Hospital admissions, Obesity, Mortality, Prevention of diabetes, Prevention of cardiovascular disease, ICD10, Kaplan–Meier, Cox proportional hazards model

## Abstract

**Background and aim:**

Aim of this retrospective study was to compare long-term mortality and incidence of new diseases [diabetes and cardiovascular (CV) disease] in morbidly obese diabetic and nondiabetic patients, undergoing gastric banding (LAGB) in comparison to medical treatment.

**Patients and methods:**

Medical records of obese patients [body mass index (BMI) > 35 kg/m^2^ undergoing LAGB (n = 385; 52 with diabetes) or medical treatment (controls, n = 681; 127 with diabetes), during the period 1995–2001 (visit 1)] were collected. Patients were matched for age, sex, BMI, and blood pressure. Identification codes of patients were entered in the Italian National Health System Lumbardy database, that contains life status, causes of death, as well as exemptions, drug prescriptions, and hospital admissions (proxies of diseases) from visit 1 to September 2012. Survival was compared across LAGB patients and matched controls using Kaplan–Meier plots adjusted Cox regression analyses.

**Results:**

Observation period was 13.9 ± 1.87 (mean ± SD). Mortality rate was 2.6, 6.6, and 10.1 % in controls at 5, 10, and 15 years, respectively; mortality rate was 0.8, 2.5, and 3.1 % in LAGB patients at 5, 10, and 15 years, respectively. Compared to controls, surgery was associated with reduced mortality [HR 0.35, 95 % CI 0.19–0.65, p < 0.001 at univariate analysis, HR 0.41, 95 % CI 0.21–0.76, p < 0.005 at adjusted analysis], similar in diabetic [HR 0.34, 95 % CI 0.13–0.87, p = 0.025] and nondiabetic [HR 0.42, 95 % CI 0.19–0.97, p = 0.041] patients. Surgery was also associated with lower incidence of diabetes (15 vs 48 cases, p = 0.035) and CV diseases (52 vs 124 cases, p = 0.048), and of hospital admissions (88 vs 197, p = 0.04).

**Conclusion:**

Up to 17 years, gastric banding is associated with reduced mortality in diabetic and nondiabetic patients, and with reduced incidence of diabetes and cardiovascular diseases.

## Background

Bariatric surgery improves quality of life in morbid obesity, prevents development of medical complications of obesity [1, 2], reduces the frequency of co-morbidities, improves cardiovascular (CV) risk profile [3–7], and is cost-effective in the management of obesity [8, 9]. A few papers [10–17], analyzed in a meta-analysis by our group [18], have described reduced long-term mortality after bariatric surgery in comparison with non-surgery controls. Even though worldwide trends in choice of surgical techniques are changing [19], the above mortality studies were performed using gastric banding (LAGB), vertical banded gastroplasty, and gastric bypass (RYGB). More recent studies have substantiated this finding showing a decreased number of CV events [20]. Studies have later shown an improved life expectancy over controls also for surgery patients suffering from CV diseases [21] and in cohorts predominantly made of male patients [22].

However, studies have limitations, such as a high rate of drop-outs (up to 40 % missing at 10 years) [11, 13, 16]. In addition, duration of follow-up was very short [11], or different for surgery patients and controls [10, 17]. Numbers of surgery patients and controls were quite different [12, 17]; the nature of control patients varied widely [15, 17], and one major concern is that control patients were simply obese patients, not patients seeking medical advice because of obesity. Some studies lacked a description of causes of death [11, 12, 16], and only two studies considered diabetic patients, one made 100 % of diabetic patients [10], the other only with 10 % of patients affected by diabetes [13]. More important, in spite of the recommendations by the International Diabetes Federation: Bariatric surgery is an appropriate treatment for people with type 2 diabetes and severe obesity (BMI ≥ 35 kg/m^2^) [23], no study compared death rates in diabetic and nondiabetic patients. A few studies also showed that bariatric surgery prevents major complications of obesity, namely diabetes mellitus and hypertension [24–28], but no study compared new incident diseases in diabetic and nondiabetic patients.

The first aim of this retrospective study was to analyze long-term mortality in diabetic and nondiabetic obese patients undergoing bariatric surgery (LAGB) in comparison with standard medical treatment, in a group of Institutions using LAGB with a common protocol. The second aim was to investigate if benefits of LAGB depend on the age of patients and controls, a possibility suggested by some previous studies [14, 16, 17, 20]. The third aim was to analyze development of co-morbidities in diabetic and in nondiabetic patients, in particular CV disease and diabetes, and therefore we looked for exemptions from medical charges (see under Methods) and hospital admissions, used as a proxy of incidence of new diseases.

## Methods

### Patients

Four Institutions (Ospedale San Paolo, Ospedale Policlinico, Ospedale San Raffaele, Istituto Clinico Sant Ambrogio, Milano, Italy) offer medical or surgical treatment of obesity, with and without diabetes. The Institutions perform bariatric surgery (LAGB) since 1995 (Ospedale San Paolo since 2001), according to NIH guidelines [27] on the basis of a common protocol [28]. We considered all obese patients (BMI > 35 kg/m^2^) aged 18–65 years, seeking medical advice, referring to the outpatients clinics of 4 institutions for obesity during the period 1995–2001, (first visit) undergoing thereafter LAGB (all Institutions) or medical treatment as outpatients (Ospedale San Paolo and Ospedale San Raffaele). The protocol was the same, and was already described [28]; as controls, we considered patients attending the obesity and diabetes outpatient clinics who refused surgery, but agreed to be followed-up. All patients were treated with diet, and received standard care (education on eating behaviors, advice on diet and exercise, plus drug treatment for diabetes when present). At least initially, all patients were evaluated under basal conditions and at 3-month intervals with measurement of body weight and assessment of food intake through review of diet diaries; their suggested diet was between 1000 and 1200 kcal/d for women and men (22 % protein, 29 % lipids, and 49 % carbohydrates), respectively, with the aid of a dietitian.

Since the beginning (June 1995), the study was intended as a possible long-term study, and two recalls were made in 2004 and in 2007 [25, 29]. The specific study protocol was approved by four Ethics Committees in 2012, after the initial protocol had been approved in 1995, in 2002 and in 2006. From the medical records, birthdate and age, anthropometric data (height, weight, BMI, systolic and diastolic blood pressure, heart rate), metabolic data (fasting blood glucose, HbA1c (%), cholesterol, HDL-, and LDL-cholesterol, triglycerides, AST, ALT, creatinine and eGFR [Modified Diet in Renal Disease Calculation Equation] [30]), current treatments, clinical evidence of coronary heart disease (CHD), retinopathy, were derived and tabulated. Diagnosis of hypertension and of diabetes mellitus was established as already reported [25, 29], and diagnosis of coronary heart disease (CHD) was based on medical records.

### Outcomes

Death rate and cause of death among diabetic patients (surgery vs no-surgery) and among nondiabetic patients (surgery vs no-surgery); exemptions and hospital admissions among diabetic and nondiabetic patients (surgery vs no-surgery). Analysis of survival and of other outcomes was carried out on the basis of initial inclusion in a group, with no consideration for LAGB removal.

### Procedures

Patients were identified through personal identification codes; codes were entered the Regional Lumbardy Administrative Database, and it was possible to ascertain whether patients were alive, were dead, or had moved to other regions. Of 1554 patients initially considered, 64 had moved outside Lumbardy Region and were not further considered. Table 1 shows patients in the study.

**Table 1:** Subjects in the study in patients matched (DM and No-DM separately) for sex, age, BMI, systolic and diastolic blood pressure (surgery vs no-surgery)

Groups	1	3	2	4
Patients (M/W) before matching	74 (16/58)	221 (101/120)**	454 (76/378)	748 (222/526)**

	DM SURG	DM No-SURG	No-DM SURG	No-DM No-SURG
Patients (M/W)	52 (15/37)	127 (36/91)	333 (78/255)	554 (136/418)
Age (years)	49.9 ± 5.25	51.9 ± 8.61	39.2 ± 10.37	40.2 ± 12.03
BMI (kg/m^2^)	43.0 ± 3.98	41.9 ± 6.31	41.1 ± 5.36	40.9 ± 7.31
Systolic BP (mmHg)	142.0 ± 12.29	148.0 ± 22.46	134.9 ± 16.48	134.4 ± 16.74
Diastolic BP (mmHg)	85.3 ± 5.95	85.9 ± 11.28	82.3 ± 10.41	82.8 ± 10.42
Heart rate (bpm)	80.9 ± 4.15	77.4 ± 3.64	75.6 ± 4.46	70.4 ± 4.72
Arterial hypertension	15	53	77	132
Creatinine (µmol/l)	74.4 ± 21.95	77.3 ± 25.26	74.6 ± 16.14	76.7 ± 19.84
eGFR (ml/min/1.73 m^2^)	88.8 ± 20.19	88.0 ± 24.87	92.1 ± 26.13	87.8 ± 18.67
BG (mg/dl)	169.7 ± 60.56	185.3 ± 62.55	92.4 ± 12.69	94.7 ± 12.19
DM drug treatment	6	27		
HbA1c (%)	7.2 ± 2.19	8.1 ± 1.88	5.7 ± 1.17	5.6 ± 1.25
Total cholesterol (mg/dl)	218.7 ± 43.74	219.4 ± 55.33	210.4 ± 43.26	212.0 ± 98.31
HDL-cholesterol (mg/dl)	50.3 ± 14.71	46.8 ± 13.46	51.1 ± 12.94	50.0 ± 15.10
LDL-cholesterol (mg/dl)	147.6 ± 38.43	144.4 ± 45.23	136.0 ± 37.24	140.2 ± 99.26
Triglycerides (mg/dl)	159.3 ± 80.06	208.8 ± 198.23	127.5 ± 70.62	135.3 ± 68.88
AST (U/l)	30.9 ± 22.28	30.6 ± 22.30	22.8 ± 10.20	24.6 ± 12.44
ALT (U/l)	42.3 ± 31.11	44.6 ± 44.93	31.2 ± 22.70	33.9 ± 22.67
Retinopathy	1	7		
CHD	0	13*	4	22*

Mean ± SD or absolute frequencies

*DM* diabetes mellitus, *SURG* surgery, *No*-*DM* nondiabetic, *No*-*SURG* non-undergoing surgery, *BP* blood pressure, *eGFR* estimated glomerular filtration rate, *FBG* fasting blood glucose, *CHD* coronary heart disease

* p < 0.05 surgery versus no surgery; ** p < 0.001 surgery versus no-surgery

The National Health System covers more than 95 % of all hospital admissions, medical and surgical procedures and medical expenses of citizens [31] (Italian Survey 2012). A Regional Lumbardy Administrative Database contains since 1988 all pertinent data of all citizens, and this makes life status a clear finding, independently of participation in studies and of loss to follow-up. In particular, the Lumbardy database collects several informations, including (1) an archive of residents who receive NHS assistance, reporting demographic and administrative data; (2) a database on diagnosis at discharge from public or private hospitals of the region; (3) a database on outpatient drug prescriptions reimbursable by the NHS; and (4) a database on outpatient visits, including visits in specialist ambulatory care and diagnostic laboratories accredited by the NHS. For each patient, these databases are linked through a single identification code. Full details of the procedures are reported elsewhere [32].

In the Italian National Health System development of chronic diseases (diabetes mellitus, liver and cardiovascular diseases, selected thyroid, renal, and lung diseases) yields the right to exemption from medical charges (exemptions), that means life-long free prescriptions and examinations for the above diseases. Therefore, together with hospital admissions, exemptions were considered a proxy of development of chronic diseases. For each patient, exemptions and hospital admissions after first visit were identified and dated. Through registries of surgeons and the Regional Lumbardy Administrative Database it was also possible to retrieve patients who had removal of LAGB and/or new bariatric surgery procedures. Through the health districts (ASL) patients belonged to, it was possible to track causes of death, and nature of hospital admissions and of exemptions. Data from health districts were cross-checked with data from the Regional Lumbardy Administrative Database, to rule out inconsistencies and possible delays in transcriptions. This procedure has already been validated in many researches [32–37]. The limit date of September 30, 2012 was established for all patients for deaths, admissions, and exemptions. Causes of death, as well as exemptions and hospital admissions were coded according to ICD-10 codes.

### Statistical analysis

Data are shown as average values (±SD) for continuous variables or absolute numbers and frequencies for discrete variables. Continuous variables were compared with the Student’s *t* test. Frequencies were compared with the Fisher exact test. The median age of the whole cohort was 43 years. Surgery patients (diabetic and nondiabetic) were more frequently women, were younger and heavier, with lower systolic blood pressure and a lower frequency of CHD than no-surgery patients. At a preliminary analysis we found that men had a higher mortality than women (Fisher exact test: 50/415 vs 59/1082, p = 0.0001), diabetic patients had a higher mortality than nondiabetic patients (47/293 vs 62/1204, p = 0.0001), older patients had a higher mortality than younger patients (above and below the median age (93/747 vs 16/750, p = 0.0001), and patients with CHD had a higher mortality than patients without CHD (14/55 vs 95/1442, p = 0.0001). Therefore, surgery and no-surgery patients were matched (diabetic and nondiabetic patients separately), with no attempt to match patients of the whole cohort. Group matching was made for sex, BMI (±5 kg/m^2^), age (±10 years), for systolic (±5 mmHg), and diastolic (±5 mmHg) blood pressure. The median age of matched patients was 42 years, and the mean ages were 31.8 ± 6.43 and 51.8 ± 5.89, respectively.

The proportion of dying patients was plotted through Kaplan–Meier curves, and differences in survival among subgroups were tested by the log-rank test. Cox proportional hazards model was used to select significant prognostic factors; the following covariates were entered a priori: age, sex, diabetes mellitus, and presence of CHD. A multivariable analysis of risk factors for mortality was performed (Cox proportional hazards model), and used to plot Kaplan–Meier curves for surgery versus no-surgery patients. Crude Kaplan–Meier curves were plotted to compare mortality (surgery vs no-surgery patients) for diabetic and nondiabetic patients separately. Proportionality among the survival rates and attributable factors in the Cox model was assessed by plotting the log [−log (survival function)] versus time in each subgroup. Statistical analyses were performed with STATA 12.0 for Windows. This manuscript was prepared following the guidelines of the STROBE statement [38].

## Results

Table 1 shows baseline clinical and metabolic data of matched patients in the study. In total, 77 deaths were observed (12 in the surgery group vs 65 in the control group, p = 0.0001). Mortality rate was 2.6, 6.6, and 10.1 % in controls at 5, 10, and 15 years, respectively; mortality rate was 0.8, 2.5, and 3.1 % in LAGB patients at 5, 10, and 15 years, respectively. Removal of LAGB occurred in 54 patients; all of them were alive on September 30, 2012. The effect og age on mortality was highly significant, as only 10/538 deaths occurred below the age of 42, as opposed to 67/528 above the age of 42, p = 0.0001. In contingency tables, the effect of quartiles of age on mortality in no-surgery patients (3/171, 4/158, 10/163, 48/186 from 1st to 4th quartile, p = 0.001) was not significant in surgery patients (1/111, 2/95, 5/115, 4/64, p = 0.188). In addition, even though patients were matched for several factors (see above), mortality was higher in men than in women (34/265 vs 43/801, p = 0.0004), in diabetic versus nondiabetic patients (40/179 vs 37/887, p = 0.0001), and in patients with than in patients without CHD (11/39 vs 66/1027, p = 0.0001).

**Fig. 1:**
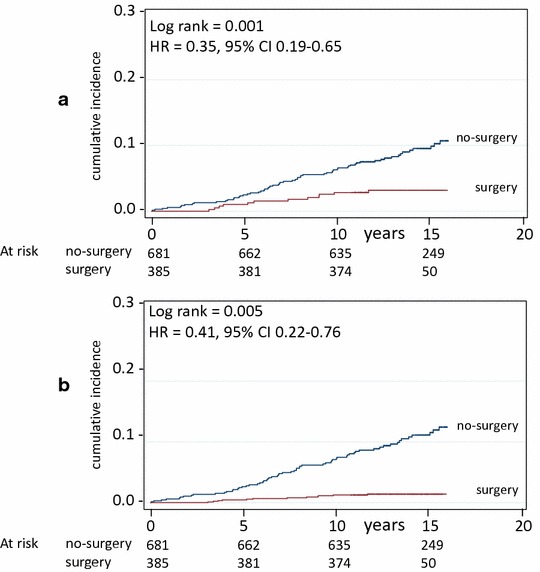
Mortality in surgery and in matched (no-surgery) control patients. Crude survival (**a**) and survival adjusted for age, sex, and for the presence of diabetes and of coronary heart disease (**b**). Number of patients at risk is indicated. Years = since visit 1

Due to the effect of age on mortality, the median age was used to model mortality curves: after adjusting for median age, sex, presence of diabetes and of CHD, Fig. 1 shows that mortality was significantly lower in surgery than in no-surgery patients both in unadjusted (HR 0.35, 95 % CI 0.19–0.65, Log rank = 0.001) and in adjusted analysis (HR 0.41, 95 % C.I. 0.22–0.76, Log rank p = 0.005). Table 2 shows results of univariate and multivariable analysis of risk of mortality in surgery versus no-surgery patients.

**Table 2:** Univariate and multivariable analysis of risk factors for mortality (Cox proportional hazards model) in the whole sample

	HR	S.E.	z	p	95 % CI
(a) Univariate analysis					
Surgery	0.35	0.11	−3.33	0.001	0.19–0.65
Age >42 years	7.15	2.43	5.81	0.001	3.68–13.91
Female sex	0.39	0.09	−4.02	0.001	0.25–0.62
Cotonary heart disease	4.67	1.52	4.73	0.001	2.47–8.86
Diabetes	5.71	1.31	7.61	0.001	3.54–8.94
(b) Multivariate analysis					
Surgery	0.41	0.13	−2.82	0.005	0.22–0.76
Age >42 years	4.35	1.57	4.08	0.001	2.15–8.82
Female sex	0.39	0.09	−4.10	0.001	0.25–0.61
Cotonary heart disease	2.51	0.83	2.75	0.006	1.31–4.81
Diabetes	3.11	0.75	4.69	0.001	1.93–4.99

Hazard ratios (HR, with 95 % CI) and standard errors are indicated, together with effect (z) and significance level

**Fig. 2:**
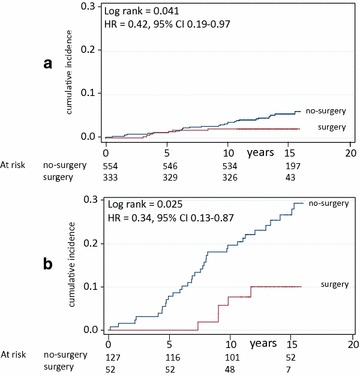
Mortality in surgery and in matched (no-surgery) control patients divided into nondiabetic (**a**) and diabetic (**b**) patients. Number of patients at risk is indicated. Years = since visit 1

Figure 2 shows that the effect was similar in nondiabetic (HR 0.42, 95 % CI 0.19–0.97, Log rank p = 0.041) and in diabetic patients (HR 0.34, 95 % CI 0.13–0.87, Log rank p = 0.025). The effect of surgery on reduction of mortality was slightly superior in diabetic (5/52 vs 35/127, p = 0.0097, i.e. 9.6 vs 28 %) than in nondiabetic patients (7/333 vs 30/554, p = 0.01569, i.e. 2 vs 5 %), but the difference was not significant.

Causes of mortality are indicated in Table 3; total deaths, deaths from cardiovascular (CV) causes, from all non-CV causes, and from neoplasia were significantly less in surgery than in no-surgery patients. No significant differences were found when subdividing diabetic and non-diabetic patients, likely due to the rather small number of deaths. Baseline BMI, HbA1c, and heart rate were not important in predicting mortality (HR 1.02, 95 % CI 0.98–1.04, p = 0.312; HR 0.99, 95 %CI 0.82–1.21; HR 1.02, 95 % CI 0.95–1.09, respectively). Weight loss (mean ± SE) could be measured in a total of 224 patients (100 surgery patients and 124 control patients) after a mean period of 12.1 ± 0.86 years, and was −3.99 ± 0.53 vs +1.1 ± 0.63 kg/m^2^, p < 0.001.

**Table 3:** Causes of death in surgery versus no surgery patients

Group	Surgery	No-surgery	Significance, *p*
CVD causes (ICD I00–I98)	5	22	0.0014
Total non-CVD causes	7	43	0.0007
Neoplasia (ICD C00–D49)	7	33	0.0116
Liver diseases (ICD K00–K92)		4	
Lung diseases (ICD J00–J98)		3	
Infection (ICD A00–B99)		3	
Total	12	65	0.0001

No significant differences were found when subdividing diabetic and nondiabetic patients in surgery versus no-surgery patients

Table 4 shows that exemptions from medical expenses occurred more for no-surgery than surgery patients; this was statistically significant for the total number of exemptions, for CV diseases, and for type 2 diabetes; considering separately diabetic (surgery vs no-surgery) and nondiabetic (surgery vs no-surgery) patients, differences were significant for total number of exemptions and for CV diseases in nondiabetic patients. New exemptions for arterial hypertension (ICD I10-I15) were 42 and 107 in surgery and in no surgery patients, respectively; therefore, the final figures of hypertensive patients were 134 surgery versus 251 no-surgery (p < 0.05). Frequency and type of exemption were not different in diabetic and nondiabetic patients. Hospital admissions were less frequent in surgery than in no-surgery patients (p = 0.04), but none individual ICD reached statistical significance (Table 5).

**Table 4:** Exemptions from medical expenses after initial visit in surgery versus no-surgery patients and in the four groups of patients

	Exemptions		*p*	DM surgery	DM no-surgery	NO-DM surgery	NO-DM no-surgery
	Total surgery	Total no-surgery					
Diabetes mellitus^a^							
ICD E10–E14	15	48	0.035	0	0	15^§^	48
CVD diseases							
ICD I00-I98	52	124	0.048	12	26	40^§^	98
ICD I10–I15	42	107	0.034	10	21	32	86
ICD I20–I25	10	17		2	5	8	12
Liver diseases							
ICD K00–K92	8	18	NS	4	7	4	11
Neoplasia							
ICD C00–D49	4	17	NS	2	5	2	12
Lung diseases							
ICD J00-J98	4	9	NS	1	4	3	5
Renal diseases							
ICD N00–N99	0	3	NS	0	2	0	1
Metabolic, diseases							
ICD 10 E70–E90	4	13	NS	1	6	3	7
Total	87	232	0.001	20	50	67^§§^	182

*DM* diabetic patients, *NO*-*DM* nondiabetic patients

^§^p < 0.05 versus no-surgery; ^§§^ p < 0.001 versus no-surgery (DM surgery vs DM no-surgery; no-DM surgery vs no-DM no-surgery)

^a^For diabetes mellitus exemptions only non diabetic patients at baseline are considered (333 and 554, respectively)

**Table 5:** Hospital admissions after initial visit in surgery versus no-surgery patients

Hospital admissions	Total surgery	Total no-surgery	p
Diabetes mellitus			
ICD E10–E14	14	33	NS
CVD diseases			
ICD I00–I98	34	69	NS
Liver diseases			
ICD K00–K92	4	11	NS
Neoplasia			
ICD C00–D49	9	22	NS
Lung diseases			
ICD J00–J98	5	18	NS
Renal diseases			
ICD N00–N99	0	4	NS
Metabolic, diseases			
ICD 10 E70–E90	5	12	NS
Muscular and bone diseases			
ICD M00–M99	17	28	NS
Total	88	197	0.04

## Discussion

To our knowledge, this is the longest follow-up study peformed to investigate mortality in LAGB as opposed to medical treatment. We found that after a follow-up period of up to 17 years (mean 13.9 ± 1.87 years), diabetic and nondiabetic patients undergoing LAGB died less frequently than control patients, and experienced a lower number of new diseases, in particular CV diseases and diabetes, as indicated by exemptions for chronic diseases and hospital admissions. Death counts were similarly lower, for both diabetic and nondiabetic patients, with surgey than in controls; in contrast, for exemptions, when considering diabetic and nondiabetic patients separately, a significant benefit (surgery vs no-surgery), appeared only for the latter.

Reduced mortality applied to both CV mortality and to all-cause mortality, in particular to neoplasia-induced mortality; other causes of death were too few to make any comparison meaningful. Reduction of mortality was similar in diabetic and nondiabetic patients, as already reported in the SOS study with a shorter follow-up period, in a cohort in which diabetic patients accounted for only 10 % [13, 20]. The benefit shown in this study (HR 0.35, 95 % CI 0.19–0.65 at univariate analysis; HR 0.41, 95 % CI 0.22–0.76 at multivariate analysis) was similar to the average benefit observed in shorter studies (meta-analysis) [18], and to previous studies in which LAGB was used [14, 17]. In addition, the benefit appeared for patients aged ≥42 years, as suggested by previous studies [14, 16, 17, 20]. This is likely due to the low mortality rate of younger patients; for instance, in the SOS study, patients under the age of 37 years were excluded to ensure high overall mortality [13]. Similar to SOS study [39], we also found a significant reduction of neoplasia, only in neoplasia-induced mortality, not in neoplasia-induced hospital admissions or exemptions.

This study has strengths and limitations. This was not a purely administrative study, as we identified obese patients seeking medical advice at the four Institutions; both diabetic and nondiabetic patients were from the same cohort, asking for medical advice, and either undergoing medical treatment or surgery; therefore, we dealt with patients that were not simply obese. At the same time, dealing with outpatients, we avoided the possible bias of patients hospitalized for serious diseases; this, in our opinion, is representative of obese patients. Looking at medical baseline records together with administrative records offers a greater number of variables (anthropometric and clinical data, biochemistry) than using administrative records alone.

In addition, this is the longest follow-up study performed so far, with no patient lost to follow up; the low number of patients observed after a mean period of almost 14 years simply depends on the late beginning of treatment, surgical or medical, and a longer follow-up period will make these numbers greater. Also, the results were obtained after matching patients for age, sex ratio, BMI, systolic and diastolic blood pressure. A similar matching has been used in similar, albeit shorter-duration studies [13, 20, 22], while in other studies matching had been done only for age, sex, and BMI [11, 12, 14, 15]. Since this is an ongoing study, a further 5 years follow-up study will answer many of the as yet unresolved questions.

The limitations lie in the relatively small number of patients. Second, this study was carried out in Institutions that offered medical or surgical treatment with the indications/contra-indications of that era; for instance, randomization of patients to surgery or to medical treatment was deemed unethical. Third, even though weight loss has not been routinely reported in previous studies [18], weight loss could be retrieved only in a small group of patients after a mean period of 12 years; a small but clear difference was found, similar to previous studies of shorter duration [13, 14]. Fourth, this was an analysis based on initial inclusion of patients in a group, and therefore we did not consider LAGB removal, occurring in 54 patients; no patient underwent new bariatric procedures. The fifth limitation is that of possible under-reporting of exemptions from medical expenses, but one should consider that exemptions are of significant monetary advantage for patients. We can assume that patients undergoing surgery were more concerned about their health conditions, and we can assume that if any, they were more likely to ask for exemptions than controls; this would lead to over-reporting for surgery than for no-surgery patients, and therefore for more exemptions among surgery than no-surgery patients, contrary to actual figures.

A final word of caution lies in the use of medical records: medical records were considered only at baseline, not later, even because of the high rate of loss to follow-up commonly observed in obese patients. This can lead to non-consideration of possible risk factors for mortality during the following period; for instance, atrial fibrillation [40] and high heart rate [41, 42] are common in obesity and in diabetes, and are both risk factors for mortality; even though atrial fibrillation was present in very few patients at baseline, we can not exclude that atrial fibrillation can have appeared later in our patients; on the other side, baseline heart rate did not affect mortality (being somewhat higher in surgery than in no-surgery patients). Also, baseline HbA1c did not affect mortality, but we do not know how HbA1c changed during the following years.

Benefits of bariatric surgery are probably more than simply related to improved metabolic control; improvement of metabolic control with resolution of diabetes can last several years [43], but diabetes can re-appear after resolution, while other effects (lipid metabolism, kidney function, systolic and diastolic blood pressure) can be maintained for longer periods, being instrumental in the better overall prognosis [44]. A few, mostly uncontrolled, studies have shown additional effects: improved endothelial function, decrease of intima-media-thickness (IMT), reduction of insulin resistance, decrease in vascular and general inflammation, increase of HDL cholesterol, decreased sympathetic activity, decreased left ventricular hypertrophy; these effects have recently been reviewed [45], and might explain the effects of bariatric surgery on cardiovascular disease. We found that a small weight loss persists years after LAGB, and might be one of the reasons for decreased mortality [44], together with reduced incidence of diabetes and CVD; also, the number of patients with arterial hypertension (with or without organ damage, Table 4, ICD I10-I15), was different at the end, and can be of clinical relevance.

Finally, we should remember that at present data about reduced long-term mortality after bariatric surgery are available only for LAGB and RYGB, while there is no information available for sleeve gastrectomy, biliopancreatic diversion, and biliointestinal bypass, three types of very effective surgery.

In conclusion, these data show that LAGB is associated with lower mortality up to 17 years in diabetic and nondiabetic obese patients, and with fewer new cases of diabetes and of CV disease. A new examination is planned for September 2017. If the above assumptions are correct, we expect an even greater preventive effect of LAGB in diabetic and nondiabetic patients with morbid obesity. The fact that benefits were similar in diabetic and nondiabetic patients is of clinical relevance; diabetologists should inform their patients of the potential benefits of bariatric surgery, not only the possible remission of diabetes [23], but also the protective role against excess mortality.
